# Paternal lineage early onset hereditary ovarian cancers: A Familial Ovarian Cancer Registry study

**DOI:** 10.1371/journal.pgen.1007194

**Published:** 2018-02-15

**Authors:** Kevin H. Eng, J. Brian Szender, John Lewis Etter, Jasmine Kaur, Samantha Poblete, Ruea-Yea Huang, Qianqian Zhu, Katherine A. Grzesik, Sebastiano Battaglia, Rikki Cannioto, John J. Krolewski, Emese Zsiros, Peter J. Frederick, Shashikant B. Lele, Kirsten B. Moysich, Kunle O. Odunsi

**Affiliations:** 1 Departments of Biostatistics and Bioinformatics, Roswell Park Cancer Institute, Buffalo NY, United States of America; 2 Gynecologic Oncology, Roswell Park Cancer Institute, Buffalo NY, United States of America; 3 Cancer Prevention and Control, Roswell Park Cancer Institute, Buffalo NY, United States of America; 4 Cancer Genetics and Genomics, Roswell Park Cancer Institute, Buffalo NY, United States of America; 5 Center for Immunotherapy, Roswell Park Cancer Institute, Buffalo NY, United States of America; Cleveland Clinic Genomic Medicine Institute, UNITED STATES

## Abstract

Given prior evidence that an affected woman conveys a higher risk of ovarian cancer to her sister than to her mother, we hypothesized that there exists an X-linked variant evidenced by transmission to a woman from her paternal grandmother via her father. We ascertained 3,499 grandmother/granddaughter pairs from the Familial Ovarian Cancer Registry at the Roswell Park Cancer Institute observing 892 informative pairs with 157 affected granddaughters. We performed germline X-chromosome exome sequencing on 186 women with ovarian cancer from the registry. The rate of cancers was 28.4% in paternal grandmother/granddaughter pairs and 13.9% in maternal pairs consistent with an X-linked dominant model (Chi-square test X2 = 0.02, p = 0.89) and inconsistent with an autosomal dominant model (X2 = 20.4, p<0.001). Paternal grandmother cases had an earlier age-of-onset versus maternal cases (hazard ratio HR = 1.59, 95%CI: 1.12–2.25) independent of BRCA1/2 status. Reinforcing the X-linked hypothesis, we observed an association between prostate cancer in men and ovarian cancer in his mother and daughters (odds ratio, OR = 2.34, p = 0.034). Unaffected mothers with affected daughters produced significantly more daughters than sons (ratio = 1.96, p<0.005). We performed exome sequencing in reported BRCA negative cases from the registry. Considering age-of-onset, one missense variant (rs176026 in *MAGEC3*) reached chromosome-wide significance (Hazard ratio HR = 2.85, 95%CI: 1.75–4.65) advancing the age of onset by 6.7 years. In addition to the well-known contribution of BRCA, we demonstrate that a genetic locus on the X-chromosome contributes to ovarian cancer risk. An X-linked pattern of inheritance has implications for genetic risk stratification. Women with an affected paternal grandmother and sisters of affected women are at increased risk for ovarian cancer. Further work is required to validate this variant and to characterize carrier families.

## Author summary

Our article uses the largest familial study of ovarian cancer to argue that there exists an ovarian cancer susceptibility gene on the X-chromosome acting independently of BRCA1 and BRCA2. This observation implies that there may be many cases of seemingly sporadic ovarian cancer that are actually inherited; for example, only daughters who inherit risk from their fathers. This X-linked pattern implies novel ways to prioritize families for screening even without additional testing—sisters must both be carriers or neither; fathers of women with potentially inherited ovarian cancer may receive new attention. In addition, we found evidence that other cancers affect fathers and sons in these families. Using sequencing technology, we isolated a candidate gene, MAGEC3, that may be associated with earlier onset of ovarian cancer. The further study of this gene and the X-linked pattern will require additional study.

## Introduction

A history of ovarian cancer among first-order relatives remains the strongest and best-characterized predictor of ovarian cancer risk [1–3] and a main determinant of genetic testing referral [4, 5]. The evidence for a monogenic, autosomal dominant mode of inherited risk dates to the pre-BRCA era where studies focused on assessing heritability [6,7] using affected first-order and second-order [8] *female* relatives. In a systematic review, Stratton and colleagues noted that, “not explicable in terms of any genetic model,” an affected woman’s sisters are at higher risk of disease than their mother [1]. We propose an explanation to this paradox is the existence of an X-linked gene that must pass preferentially from a carrier father to each of his daughters.

Genetic evidence of X-linkage has appeared in cytogenetic studies where loss of X-chromosome inactivation (XCI) can be visualized by loss of heterochromatin based Barr bodies [9]. Studies of ovarian tumors’ genomic profiles show loss of heterozygosity around Xq25 and Xp [10,11] as well as patterns of XCI [12,13] possibly associated with tumors of low malignant potential [14]. Studies investigating a mechanistic connection between *BRCA1* and XCI [15,16], especially in tissue after transformation [17], are mixed but tend to conclude that XCI dysregulation is BRCA independent [9,18,19].

**Fig 1:**
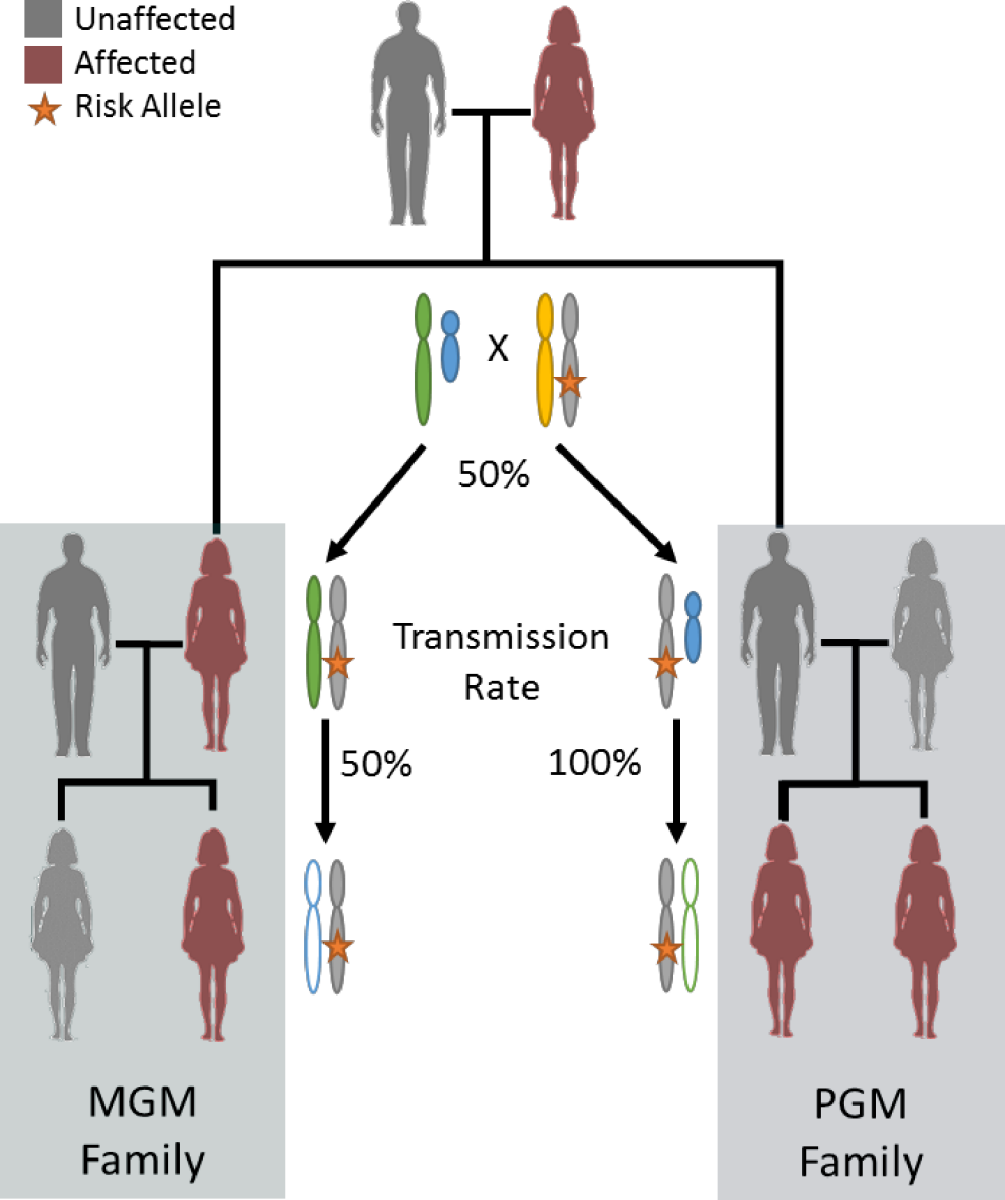
X-linked model. Schema for X-linked inheritance when cancer status is specific to women (all carrier men are effectively disease censored). Two family patterns with a pair of first-degree affected women are the maternal grandmother (MGM) family and the paternal grandmother (PGM) family. Stratton’s paradox implies that PGM families are more likely under X-linkage because a father must pass the variant to all of his daughters. The rates are equal if the variant is autosomal.

The Familial Ovarian Cancer Registry housed at Roswell Park Cancer Institute (Buffalo, NY), for over 35 years comprises over 50,000 participants and 5,600 cancers in 2,600 families. To leverage the deep pedigree data in this study, we reasoned that, if the disease allele passes through the father’s side of the family, it could be inferred by disease in a woman’s father’s mother. That is, by considering the frequency of disease transmission in grandmother/granddaughter pairs with an intermediate son/father. Under an autosomal dominant model, an affected grandmother (either maternal or paternal) passes the disease allele to her granddaughter with probability 1/4. This means that data previously presented by affected mothers and sisters are not able to discriminate between autosomal and X-linked models and these effects have been previously indistinguishable by segregation analysis due to disease censoring in fathers. In the X-linked dominant model, while a maternal grandmother again passes the disease allele to her granddaughter with probability 1/4, a paternal grandmother passes the allele to her granddaughter with probability 1/2 due to deterministic transmission by the obligate carrier son/father. Therefore, we might discriminate between autosomal versus X-linked models by considering the rate of cancers in granddaughters with exactly one affected grandmother. An autosomal dominant model predicts an equal rate of cancers in maternal-lineage and paternal-lineage pairs while an X-linked dominant model predicts that paternal-lineage families will have twice the rate of cancers (**Fig 1**).

We collected almost 3,500 grandmother/granddaughter pairs within the registry to test this paternal lineage hypothesis and were able to sequence 159 germlines to search for candidate variants.

## Results

### First degree relative ovarian cancer risk

We recapitulated Stratton’s paradox as outlined in Table 1: while mothers of affected women had a high risk of cancer (35%, 95% confidence interval (CI): 33–37%), sisters had a further elevated risk (66%, 65–67%). Unaffected women yielded an increased maternal risk (24%, 23–26%) while their sisters had the lowest risk (15%, 15–16%). The relative risks (RR) in the registry study (mothers RR = 1.43, sisters RR = 4.42) are close to Stratton’s original estimates (mothers RR = 1.1, sisters RR = 3.6) [1]. The effect remained for the eldest daughter in each family versus her sisters, obviating correlation due to multiple sister-pairs. We also noted that these effects were robust to stratification by familial BRCA status and families manifesting a hereditary breast and ovary pattern of disease versus families with site-specific ovary disease.

**Table 1:** Cancer rates given an affected daughter or sister.

	Given daughter/sister has				
Ovarian cancer risk ^a^	No Cancer	Ovarian Cancer	N pairs	Relative Risk	Odds
					Ratio
Whole Registry					
Mother	24% (23–26%)	35% (33–37%)	8909	1.43 (1.34–1.52)	1.66 (1.51–1.82)
Sister	15% (15–16%)	66% (65–67%)	27133	4.42 (4.25–4.58)	11.17 (10.52–11.87)
BRCA Positive Family					
Mother	23% (20–26%)	29% (25–34%)	1208	1.29 (1.06–1.58)	1.41 (1.07–1.86)
Sister	15% (14–16%)	59% (56–62%)	3938	4.00 (3.62–4.42)	8.37 (4.07–3.86)
BRCA Negative Family					
Mother	22% (19–26%)	35% (30–41%)	773	1.59 (1.26–2.00)	1.91 (2.07–7.86)
Sister	18% (16–20%)	61% (57–64%)	2383	3.36 (2.98–3.78)	6.89 (3.07–2.86)
Breast and Ovary Family					
Mother	28% (27–30%)	41% (38–44%)	3430	1.44 (1.31–1.58)	1.74 (1.07–3.86)
Sister	17% (16–18%)	68% (67–70%)	9408	3.89 (3.75–4.22)	10.42 (4.07–9.86)
Site-Specific Ovary Family					
Mother	33% (31–36%)	44% (41–46%)	2966	1.31 (1.19–1.43)	1.54 (1.07–2.86)
Sister	18% (17–19%)	69% (67–70%)	10797	3.78 (3.54–3.94)	9.73 (3.07–1.86)

^a^ All individuals without cancer are dead without disease or older than 45.

### X-linked genetic model testing

Among granddaughters with one affected grandmother (Table 2), we observed a paternal-lineage cancer rate of 28.4% (95% CI: 22.8–34.8%) and maternal-lineage cancer rate of 13.9% (11.4–16.8%). The paternal-lineage women had 2.04 times the risk (1.55–2.71) of maternal-lineage women, consistent with the X-linked dominant model that assumes the rate of paternal-lineage cancers is twice the maternal-lineage rate (goodness-of-fit, chi-square X^2^ = 0.2, p = 0.89). The autosomal dominant model predicted too many maternal cancers and too few paternal cancers (X^2^ = 20.4, p<0.001). The X-linked effect was robust versus ascertainment bias; we repeated the analysis excluding the granddaughters who were probands and observed a nearly identical relative risk (RR = 2.03, 1.28–3.23).

**Table 2:** Rates of granddaughter cancer in grandmother-granddaughter pairs.

	Registry women with exactly 1 grandmother with ovarian cancer		
	Mother’s Mother	Father’s Mother	
All Complete Pairs			
Observed Pairs	663	229	
Granddaughter Cancer	92	65	
Cancer Rate	13.9%	28.4%	RR = 2.04
(95% CI)	(11.4–16.8%)	(22.8–34.8%)	(1.55–2.71)
Expected # Cancers			
Autosomal Dominant	116.7	40.3	X^2^ = 20.3, p<0.001
X-linked Dominant	92.5	63.9	X^2^ = 0.02, p = 0.89
Probands Excluded			
Observed Pairs	374	133	
Granddaughter Cancer	36	26	
Cancer Rate	9.6%	19.5%	RR = 2.03
(95% CI)	(6.9%-13.2%)	(13.4%-27.5%)	(1.28–3.23)
Granddaughter ≥ 45 Years^a^			
Observed Pairs	344	154	
Granddaughter Cancer	92	65	
Cancer Rate	26.7%	42.2%	RR = 1.58
(95% CI)	(22.2–31.8%)	(34.4–50.4%)	(1.22–2.04)
Site-specific Ovary Family			
Observed Pairs	291	127	
Granddaughter Cancer	49	44	
Cancer Rate	16.8%	34.6%	RR = 2.06
(95% CI)	(12.8–21.8%)	(26.6–43.7%)	(1.45–2.92)
Breast and Ovary Family			
Observed Pairs	357	89	
Granddaughter Cancer	43	21	
Cancer Rate	12.0%	23.6%	RR = 1.96
(95% CI)	(8.9–16.0%)	(15.5–34.0%)	(1.23–3.13)

RR, Relative risk; X^2^, Chi-square goodness of fit statistic.

^a^ Granddaughters without cancer who are younger than 45 are omitted

### Age-of-onset analysis

We observed a significant acceleration in the development of disease in granddaughters with an affected paternal grandmother versus maternal grandmother (log-rank test p = 0.009; hazard ratio HR = 1.59, 95% CI: 1.12–2.25). Granddaughters with an affected maternal grandmother were not more likely to manifest early onset disease versus women with two unaffected grandmothers (N = 2293, Log-rank p = 0.87, HR = 0.97, 95%CI: 0.75–1.24). While it does not affect the paternal/maternal effect conclusion, the subset of grandmother-granddaughter pairs possesses a higher risk than the average woman in the registry reflecting the selection bias towards families with more genetic follow up.

### Implications of paternal and X-linked cancer

We considered whether men in the path of transmission were more likely to develop other cancers. In the grandmothers-granddaughter trios with affected granddaughters, the intermediate father was more likely to report a prostate cancer diagnosis if his mother had had ovarian cancer (OR = 2.34, 95%CI: 1.07–5.06, Fisher's exact test p = 0.0336) implying that the three generation pattern—ovary, prostate, ovary—was unusually common.

Because cancer-causing germline variants on X might affect the fitness of offspring and lead to an imbalance in sex birth ratio, we examined whether families were more likely to report female offspring. Removing the probands from the analysis, among BRCA negative families, there was a strong bias towards producing daughters when their mother was unaffected (female to male ratio = 1.96, difference from 1.0, p < 0.0001), putatively due to an allele transmitted by the father. The effect was attenuated for affected mothers but still significantly favored daughters (ratio = 1.21, p = 0.0131). Conservatively, this number establishes a baseline for reporting bias in these families. Among *BRCA1* mutation carriers, when the mother was unaffected thereby favoring paternal transmission, there was a strong bias towards daughters (ratio = 1.19, p = 0.005). Among *BRCA1* mutation families there was no difference in sex ratio when the mother was affected (ratio = 1.07, p = 0.562). This number is close to the expected population ratio suggesting minimal ascertainment bias. There were insufficient *BRCA2* carrying families to make an assessment.

Among children of men who reported cancers other than prostate cancer, the sex ratio was consistent with no sex bias (ratio = 1.05, p = 0.554) and, while men with prostate cancer reported an excess of daughters (ratio = 1.12, p = 0.09), the effect was shy of statistical significance.

### Registry-wide pedigree analysis

To estimate the potential impact of X-linked disease, we evaluated the kinship-based likelihood for autosomal dominant and X-linked models [20] for 1,386 registry pedigrees with at least two confirmed cases of ovarian cancer. Of these pedigrees, 14 (1.0%) clearly ruled out X-linked disease due to father-son transmission while 566 (40.8%) were equally likely under X-linked or autosomal likelihood models. A quarter of families slightly favored X-linkage (338, 24.4%) and 468 (33.7%) had a likelihood ratio greater than 1.5.

**Fig 2:**
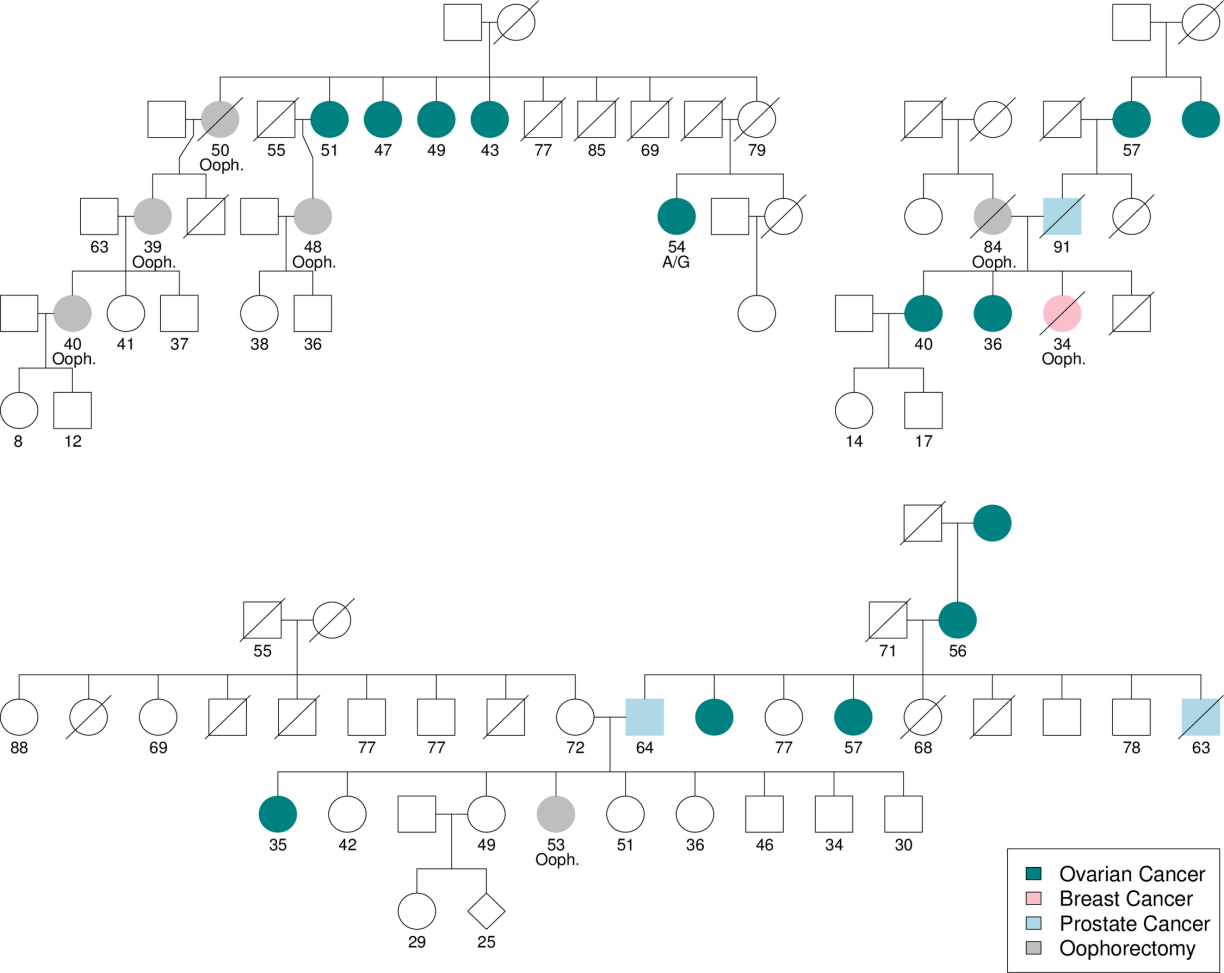
Representative registry pedigrees. Ovarian cancers are represented by teal circles, breast cancers by pink circles, prostate cancers by blue squares and prophylactic oophorectomies by grey circles. The earliest of age of onset, prophylaxis, death or last follow up is indiciated below individuals as well as oophorectomies (ooph.) and one heterozygous variant carrier (A/G).

We illustrated this imbalanced likelihood in the pedigrees represented in **Fig 2**, where in the first family, 4 of 5 daughters at risk developed ovarian cancers between the ages of 43 and 51 (a sixth daughter underwent prophylactic oophorectomy). This situation favors X-linkage: assuming a carrier penetrance of 0.65 [21] and a non-carrier penetrance of 0.15 (the rate of familial ovarian cancer), the likelihood of this observation is 0.077 under the autosomal model and 0.315 under the X-linked model; a likelihood ratio of 4.10. This pattern is therefore apparent in cases of strong familial aggregation within a generation and could be inferred probabilistically. The remaining pedigrees show the paternal grandmother/granddaughter genetic logic, selecting for pedigrees where the intervening male developed prostate cancer.

### X-chromosome exome sequencing

To identify candidate X-linked loci, we sequenced the germline X-chromosome exome and *BRCA1* coding region for 159 affected registry women who reported a negative BRCA test. The set comprised 49 cases with an affected mother only, 46 with an affected sister only, and 7 with both. Among the 2,161 common variants, one exceeded the chromosome-wide significance level (**S1A Fig**) at position ChrX:140,983,127 (GRCh37/hg19) with LOD = 4.91. This position mapped to rs176026, a missense SNP (Q8TD91, p.A328T) in the *MAGEC3* gene on Xq27.2.

We observed 138 ovarian cancer cases with the A/A genotype, 20 women with the A/G genotype and 1 woman with the G/G genotype (Hardy-Weinberg Equilibrium (HWE), X^2^ = 0.086, p = 0.96) (Table 3). While the observed minor allele frequency of 6.9% (22 of 318 alleles) was not higher than the HapMap CEU frequency (5.3%, t-test p = 0.24), the frequency of A/G genotypes was significantly higher in registry women (12.6% versus 5.3%, p<0.001). Among carriers, serous histologies were common (15/21, 71%) and we observed other common epithelial types (2 endometrioid, 2 mucinous, 1 clear cell) as well as a granulosa cell tumor; these frequencies are similar to the overall distribution of ovarian cancer histologies. Women with A/A genotypes had a median age-of-onset of 50.3 years (95%CI: 47.8–52.9) reflecting the selection for family history. Women with A/G genotypes were significantly younger at diagnosis (43.6 years, 95%CI: 40.0–48.1; log-rank p<0.001, **S1B Fig**). Notably, all women with A/G genotypes developed cancer before age 53. The A/G effect was pronounced for women with affected sisters only (HR = 6.86, 2.18–21.56) with a median 11.0 years earlier onset (p<0.001). While women who either carry the rs176026 risk allele or were from *BRCA1* variant families had similar age-of-onset (43 years), rs176026 carriers without *BRCA1* variants had a stronger hazard (HR = 3.17, 1.80–5.58, p<0.001) suggesting that the X-linked variant has the stronger effect in these families.

**Table 3:** Age-of-onset by genotype and familial pattern.

	Count	Median Age at Onset (95% CI)	Hazard Ratio (95% CI)
**rs176026**†			
A/A	138	50.3 (47.8–52.9)	Reference
A/G	20	43.6 (40.0–48.1)	2.86 (1.73–4.70)
G/G	1	—	—
**rs176026, Family BRCA status**			
A/A, Negative	127	50.9 (47.9–54.1)	Reference
A/A, BRCA1+	11	43.8 (42.2-N/A)	1.48 (0.80–2.75)
A/G, Negative	15	43.4 (37.8–49.5)	3.18 (1.81–5.59)
A/G, BRCA1+	5	43.9 (41.3-N/A)	2.43 (0.98–6.03)
**rs176026, Sister Only** †			
A/A	39	52.8 (50.3–59.5)	Reference
A/G	4	41.8 (29.9-N/A)	6.86 (2.18–21.56)
**rs176026, Mother Only** †			
A/A	36	49.6 (46.0–55.1)	Reference
A/G	7	43.4 (40.0-N/A)	3.90 (1.57–9.69)
rs176026, Neither*^,^†			
A/A	52	46.8 (41.3–52.0)	Reference
A/G	7	41.7 (34.4-N/A)	1.71 (0.76–3.84)

N/A: Upper confidence limit for median not estimable. Mother and Sister families omitted (all are A/A). Counts include 14 unaffected family members (all are A/A) and exclude women with unknown age-of-onset.

* Women are suspected to have hereditary cancer via a daughter or higher degree relative.

† Results for G/G patient suppressed.

### In silico functional analysis of rs176026

The candidate SNP was in strong linkage with rs176024 (R^2^ = 0.7395, D’ = 1.0) another misssense SNP 63 base pairs away. Within the MAGEC3 coding sequence, two intronic SNPs (rs73577987, rs73577990) were also weaky linked to rs176026 (R^2^ = 0.1153, D’ = 0.3395 and R^2^ = 0.3103, D’ = 0.6478) and retained an age of onset effect. Altogether 28 women had a variant in any of rs176026 (N = 21), rs176024 (N = 14), rs73577987 (N = 18), or rs73577990 (N = 11) with a median age of onset of 53.4 (95%CI: 50.4–57.9, log-rank p<0.0001), more than 9.4 years earlier than the rest of the familial cases (HR = 2.8, 95%CI = 1.8–4.3). The frequency of the possible haploblock (rs73577987-A, rs73577990-C, rs176024-G, rs176026-G) was 1.96% (15/766) in all European populations, 0.7% (7/1003) in all African populations, and 26.8% (205/764) in all East Asian populations.

HaploReg (v4) analysis noted that the rs176026 alternate allele increase the binding affinity for a *HOXA13* motif and a PU.1 (*SPI1*) motif. rs176024 directly affects a predicted ERα binding motif reducing the PWM score 80%. While intronic, rs73577987 affected a STAT motif and an EWSR1-FLI1 motif; rs73577990 affected BDP1, ERα, NRSF and PU.1 motifs. Functional prediction suggested that rs176026 was more likely to be deleterious to secondary protein structure: PolyPhen-2 predicted rs176026 to have a probably damaging effect (Polyphen score 0.980, sensitivity 0.75, specificity 0.96) [22] and to alter the tertiary structure of *MAGEC3* [23] forcing a conformational change that impedes access to the MAGE binding domains (**S1C Fig**). In contrast, rs176024 was scored benign (score 0.001, sensitivity 0.99, specificity 0.15).

### In silico functional studies of *MAGEC3*

The canonical isoform of *MAGEC3* (NM_138702.1, Uniprot: Q8TD91-1) possesses two copies of the MAGE homology domain (MHD) that defines members of the MAGE gene duplication family. Unlike other cancer-testis antigens, which are expected to have no expression in normal tissue, *MAGEC3* appears to have low to moderate expression in a range of normal tissues tested by the GTEx project [24] (**S2 Fig**). Using the classic CT antigen *MAGEA1* to provide a reference for lower limit of detection, we saw that *MAGEC3* had a median expression 83x (brain) higher than *MAGEA1*, 47.5x (blood vessel endothelium), 9.2x (ovary) (**S3 Fig**). Results were similar for classic CT antigens NY-ESO-1 (*CTAG1B*), *MAGEA3*, *MAGEC1* while in contrast CT-like antigens (NY-BR-1, OY-TES-1) showed higher expression in other tissues.

In contrast, the TCGA mRNA data the level was 1.2x higher (95%CI: 1.19–1.30, Affymetrix Human Exon 1.0 array) and 0.80x (95%CI: 0.65–0.98, RNAseq, Z-scores). Notably in the cancers, there were three groups present: the set around the unity line where MAGEC3 expression was at the limit of detection, a set left of the line (7.1% 23/306) where MAGEA1 is likely expressed, a set right of the line (12.4%, 38/306) where MAGEC3 may be expressed. Even for the fraction of tumors expressing MAGEC3, the level was 2.65 fold lower than normal tissue (t-test p = 0.0009). These patterns are consistent with the idea that *MAGEC3* may perform a tumor suppressive function like many inherited cancer genes.

As we noted, the G allele frequency for rs176026 in the CEU HapMap study was 5%; in the African population (YRI), G is the major allele with 65% frequency (**S4 Fig**). These frequencies are correlated with the IARC reported incidence of ovarian cancer (Pearson’s r = 0.557 all HapMap populations) especially when excluding the Chinese American (CHD), African American (ASW) and Mexican ancestry (MEX) populations living in the United States (r = 0.858). The 1000 genomes populations were similar (r = 0.903); notably the Japanese in Tokyo, Japan (JPT), Masaii in Kinyawa, Kenya (MKK) and Luhya in Webuye, Kenya (LWK) had unusually high incidence of ovarian cancer given their minor allele frequency.

### Investigating ascertainment bias

We considered whether the increased frequency of granddaughter cancers might be a result of ascertainment. Because two qualifying cancer cases are required to register a family, if a mother cannot contribute the case, then one may suspect the granddaughter is a qualifying case. In paternal grandmother/granddaughter pairs (N = 229), we noted that 164 granddaughters did not have cancer and could not be qualifying cases. The second qualifying case was a sister or paternal aunt in 79.1% of cases. In paternal pairs, 42.3% had an affected paternal aunt and 6.7% an affected maternal aunt, confirming evidence of specific lineage. The paternal/maternal granddaughter cancer rate was similar when stratifying pairs by initial ascertainment by mother/daughter (RR = 2.18, 95%CI:1.76–2.72, N = 220), sister/sister (RR = 2.51, 2.11–2.97, N = 376), or 2^nd^ degree (RR = 2.15, 1.56–2.93, N = 203) pair. We conclude that the ascertainment bias due to an unaffected mother is minimal and the notion that the relevant pairs represent paternal lineage is well supported.

Reflecting a maternal-lineage ascertainment bias, there were more maternal than paternal pairs (663 versus 229). Mean accession identifiers for maternal and paternal grandmothers are 10.3 and 14.9, respectively (two-sample t-test, p<0.001; medians 9 and 14), suggesting that we have preferentially contacted maternal grandmothers. The direction of this potential bias is consistent with our suspicion that paternal pairs are underreported and reduces the precision of the paternal-lineage cancer rate estimate. Granddaughters with ovarian cancer in maternal families were not older than paternal-lineage women (mean difference 1.8 years, two-sample t-test p = 0.39), but unaffected women were 4.2 years older (p<0.001). While statistically significant, this difference was not likely to be the source of a 14% increase in cancer risk.

The rate of granddaughter ovarian cancers was not different between families with and without BRCA1/2 mutations (37.3% versus 38.2%, two-sample t-test p = 0.89), therefore BRCA status cannot be a confounding variable for women ascertained for family history. Alternatively, we observed the risk- doubling effect when stratifying 864 pairs into site-specific ovary (RR = 2.06, 1.45–2.92) or breast/ovary syndrome families (RR = 1.84, 1.22–2.78); there was no confounding by a family history of breast cancer. Therefore, an X-linked gene might confer ovarian cancer-specific risk independent of BRCA-type disease.

## Discussion

We have presented evidence that there may exist an X-linked model of transmission of an ovarian cancer susceptibility gene. Our observations are supported by a large familial study and the novel use of grandmother/granddaughter pairs to observe an increased rate of cancer among paternal granddaughters, an earlier age of onset, and a bias towards families with more daughters. We sequenced the X chromosomes of a small number of registry members in order to isolate a candidate gene, but we cannot rule out the possibility that our reported variant is in linkage with the true variant. However, the segregation analysis and age of onset analyses do suggest that it is likely to lie on the X chromosome.

Future studies are warranted to confirm the identity and function of the X-linked gene that contributes to familial transmission of ovarian cancer. Limitations of our study include the case-only design, which has required us to forgo investigating common variants. While the number of pedigrees in the registry is large, unrelated case-control studies are much larger and would likely yield other potential variants. Our study population is nearly exclusively Caucasian and our results may not extend to other populations. Our exome sequencing approach focuses on the coding regions of the X-chromosome only. This design is unable to identify intragenic variants and complex rearrangements not involving exons.

Evidence of X-linkage is not inconsistent with the prevailing autosomal dominant BRCA1/2 with polygenic weak variant effects model for ovarian cancer [8]. Ramus and colleagues [21] previously noted a lack of BRCA mutations in more than 33% of families with 3 or more ovarian cancers and 35% of families with breast/ovary cancers and concluded that there is a missing susceptibility gene. In families with two cases of ovarian cancer, the rate of BRCA mutations increased from 27% with no breast cancers to 83% with two breast cancer cases suggesting that BRCA mutations may be more specific to breast cancers. Schildkraut and colleagues [25] inferred that there must exist both shared and disease-specific genes after estimating the heritable correlation between breast and ovary cancers at h^2^ = 0.48. The missing gene might be ovarian cancer specific.

We suspect that the difficulty identifying this missing heritability may be due, in part, to historically inconsistent disease definition. In our literature review, we noted that studies that ascertained patients for breast cancer first and then acquired family members with ovarian cancer only saw increased risk to mothers [26]. Indeed, aggregated breast/ovary cancer studies [27] tend to show the autosomal dominant model while studies that carefully isolate ovary cancers uncover the X-linked, sister/mother effect [1]: given a family history of breast cancer, a mother with breast cancer increases the ovarian cancer risk to her daughter (OR = 2.3) while an affected sister yields a negligible odds ratio (OR = 1.1). Conversely in families with a history of *ovarian cancer only*, a mother’s ovarian cancer raised her daughters’ ovarian cancer risk (OR = 2.3, but p>0.05) while a sister’s ovarian cancer nearly quadruples her sister’s risk (OR = 3.92) [28]. Therefore, the autosomal dominant genes may be common to both breast and ovary cancers while the X-linked gene may be ovary-specific. We emphasize that future studies should be carefully designed to isolate X-linked versus autosomal and ovary-specific versus breast-ovary associations and to distinguish sporadic and hereditary ovarian cancers. Identifying a significant X-linked contribution to familial ovarian cancer risk has implications for clinical genetics: with suspicion of paternal lineage, an affected woman’s sisters are at significantly increased risk for ovarian cancer and ought to be counseled. If the affected woman carries the X-linked gene through her father, her sisters must also be carriers. It is reasonable to conjecture that maternal-lineage bias may have affected how patients, physicians, and researchers view family history and so the X-linked pattern may imply a familial origin for ovarian cancers previously thought to be sporadic cases. In particular, if the disease transmits through the father’s side, cases manifesting in only children or a woman with only brothers may not appear overtly hereditary. Using the rate of second generation grandmother pairs, we observed twice as many affected maternal grandmothers versus paternal. Without ascertainment bias, we would have expected a balanced rate, so we might predict that, other things equal, we have missed almost two paternal cases for every observed one.

We have provided some evidence that *MAGEC3* is a potential candidate for the X-linked gene near previous linkage loci and it possesses a missense variant with large effect, rare prevalence and is associated with earlier onset. While *MAGEC3* is thought to be a cancer testis antigen, it shows some expression in normal heart, brain, fallopian tube and pituitary gland tissues suggesting that the loss of expression of *MAGEC3* plays some role in cancer formation. We have previously shown that co-expression patterns of the MAGE genes are non-random in ovarian tumors [29] and that other X-linked CT antigens (NY-ESO-1 encoded by *CTAG1B*) signaling highly aggressive tumors [30].

The only MHD-carrying yeast homologous gene (*NSE3*) binds with *NSE1* and complexes with *SMC5*/*SMC6* to repair double strand breaks via homologous recombination [31–33]. On the other hand, family members *MAGEA* [34, 35], *MAGEC2* [36], carrying a paralogous MHD, have been shown to bind to RING domain proteins to form a p53 interacting E3 ubiquitin ligase that promotes tumorigenesis. Recently, computational modeling of sex-bias in cancers affecting both sexes has identified *MAGEC3* directly as a putative X-linked tumor suppressor [37]. Reinforcing our observation that men in X-linked families may be at increased risk of prostate cancer, the cytoband housing *MAGEC3* (Xq27.2) has been previously linked to these hereditary cancers [38], raising the possibility that there is a common hereditary X-linked locus responsible for reproductive tract-specific cancers.

While not unexpected for the X-chromosome [39], the candidate SNP appeared to rest in HWE while the HapMap populations reject HWE for this SNP. Given that the population frequencies are correlated with ovarian cancer incidence, we conjecture that the general population is under selection against the G allele (and against ovarian cancer) and it is the registry population that inherits neutrally and therefore more often manifests disease. This conjecture is consistent with the observations that CT antigens, especially those on the X chromosome, are under strong positive selection [40] and that the region containing MAGEC3 shows strong inter-population difference [41]. While the latter study localized the effect to MAGEC2 and not MAGEC3, their criterion for the frequency difference was aggressively high (delta > 0.90) which would preclude the SNPs that may be still be in the middle of a soft selective sweep. That the beneficial A allele in rs176026 has not yet fixed may explain why we have found a common variant, but it may also imply that the true cancer phenotype variant is hitchhiking along with the selective pressure behind the CT antigens.

## Methods

### Familial Ovarian Cancer Registry

Families in the Familial Ovarian Cancer Registry (formerly Gilda Radner Familial Ovarian Cancer Registry, Buffalo, NY) have been accessioned continuously from 1981 to present as described previously [8,42]. Briefly, qualifying families must have (a) two or more cases of ovarian cancer, (b) one ovarian cancer with two or more other cancers or (c) an early onset (age 45) ovarian cancer and at least one other cancer. Families provide written informed consent under Roswell Park Cancer Institute protocol CIC95-27. Cases are verified by medical record and/or death certificate when required and stage and histology are verified by a registry pathologist. The registry comprises 50,401 individuals including 5,614 ovarian cancers from 2,636 unique families. Families are also classified by disease pattern: families manifesting only ovarian cancer are termed “site-specific ovary” families and families with a number of breast cancers as well as ovarian cancers are “breast and ovary” families.

### First and second order pair ascertainment

Considering women who (a) were at least 45 without disease at last contact or (b) had died without disease and those with confirmed ovarian cancer, we observed over 8,900 mother-daughter pairs and 27,000 sister-sister pairs. From large registry pedigrees, we ascertained 3,499 women with two grandmothers who possessed a recorded disease status. Of these granddaughters, 2,569 reported no affected grandmothers (73.4%), 892 had exactly one affected grandmother (25.5%) and 38 had two affected grandmothers (1.1%). These women came from large pedigrees where the average family under study has 27.3 individuals (range: 8–330). Of the 3,499 pairs, 619 belonged to high-risk families tested for BRCA mutations as previously described [8]. Families are classified as *BRCA1* and/or *BRCA2* positive if any one family member tests positive for a deleterious mutation. If every family member tests negative, the family is classified as BRCA negative.

### X-chromosome sequencing

Due to the age of the study cohort and availability of blood samples, we could not sequence all of the grandmother/granddaughter pairs. We focused on 159 women who reported a negative BRCA test and had an available DNA sample. DNA samples were whole-exome sequenced using Agilent SureSelect Human All Exome 50Mb kits v3 and v5. Raw sequence reads were aligned to the Human Reference Genome (NCBI Build 37) using the Burrows-Wheeler Aligner (BWA) [43], Picard [44] and GATK [45]. We retained variants within the X-chromosome exome with at least a 10% rate of non-reference genotypes, using the total, 2,161, to set the chromosome-wide significance threshold for the log-rank test of age-of-onset association at–log_10_(0.05/2161) = 4.636 on the log odds (LOD) scale. BRCA status was re-evaluated based on the sequencing results.

### Population genetics

Ovarian cancer incidence data was downloaded from the IARC website [46]. HapMap allele frequencies were accessed via dbSNP and 1000 genomes populations via the Phase 3 1000 genomes browser. Correlations between the incidence and allele frequency were assessed by simple linear regression. The sequenced women all self-report Caucasian ancestry which we confirmed through principal components analysis.

### Within sib-ship likelihood models

Assume that we observe W = W_n_ + W_c_ women with ovarian cancer among a sistership of N = N_n_ + N_c_ women, where the subscripts n and c refer to non-carriers and variant carriers. The likelihood of W given the probability of disease in carriers (p_c_) and non-carriers (p_n_) can be constructed by assuming that, conditional on N_n_ and N_c_, W_n_ and W_c_ are simply binomial random variables. Under an autosomal model, Nc is Binomial (N, 0.5). Under the X-linked model, P(N_c_ = N) = P(N_c_ = 0). Evaluating these likelihoods by enumerating admissible combinations of (W_n_, W_c_, N_n_, N_c_) is straightforward.

### Tissue expression data

We downloaded GTEx v7 data (https://www.gtexportal.org/) aligning reported female cases only with known tissue sample types. We normalized the *MAGEC3* RNAseq levels to *MAGEA1* levels on a per sample basis. TCGA ovary data on the quantile normalized HuEx array were downloaded from the GDAC Firehose (https://gdac.broadinstitute.org/) and cBioPortal’s normalized RNAseq Z-scores.

### Functional analysis

We examined SNP-based linkage via LDLink, LDproxy [47] using the CEU population for reference. The functional predictions for missense SNPs were scored by PolyPhen [22] and the regulation was scored by HaploReg (v4) [48] using the EUR reference and its default position weight-matrix scoring algorithms [49].

### Statistical analysis

Expected frequencies under the autosomal model were based on the pooled case frequency (157/892 = 17.6%). It can be shown that the X-linked likelihood was maximized by a granddaughter cancer rate of 14.0%. Goodness-of-fit was tested versus a chi-square distribution. Pedigree likelihoods were evaluated using the kinship2 [20] algorithms. Relative risk and odds ratio confidence intervals were computed under the log transform. Age-of-onset was defined as the shortest time to death, ovarian cancer diagnosis or prophylactic oophorectomy censored by age at last contact. The majority of granddaughter ages were observed (89.0%, 3080/3461). Risk of disease was estimated using the product-limit (Kaplan-Meier) estimate, tested with the log-rank test and hazard ratios estimated through Cox’s partial likelihood with graphical diagnostics for proportional hazards. All tests are two-sided and analyses were performed in R3.3.1 including the survival package.

## Supporting information

S1 Fig Sequencing results. X-chromosome wide exome sequencing (A) yielded a single SNP associated with earlier age of ovarian cancer onset (B). The variant in in *MAGEC3* affects the backbone between two MAGE homology domains (C) leading to a predicted conformational change and loss of function.
(TIF)

S2 Fig Raw RNAseq count data from GTEX. (A) *MAGEC1* and *MAGEC2* show classic cancer testis antigen patterns while *MAGEC3* shows moderate levels of expression in most tissues. (B) Log10 RPKM RNAseq data again shows *MAGEC3* has moderate expression in a variety of tissues.
(TIF)

S3 Fig MAGEC3 versus MAGEA1 expression in normal and ovarian tumors. Relative expression to MAGEA1, which is not expressed in normal tissue, is nearly 100x higher in brain tissue. The TCGA categories are ovarian tumors measured by array and RNA sequencing.
(TIF)

S4 Fig Population allele frequency. G/A allele relative frequencies by population and geographic location (A); CHD and ASW are the Chinese American and African American populations in Denver and southwest USA. Sorted by allele frequency and expected genotype frequency (B). Alelle frequency is correlated with national incidence of ovarian cancer in HapMap and 1000 genomes (C).
(TIF)
