# Supplementary figures and images for: Paternal lineage early onset hereditary ovarian cancers: A Familial Ovarian Cancer Registry study
Source: PLoS Genet. 2018 Feb 15;14(2):e1007194. doi: 10.1371/journal.pgen.1007194 (PMC5813894; doi:10.1371/journal.pgen.1007194)

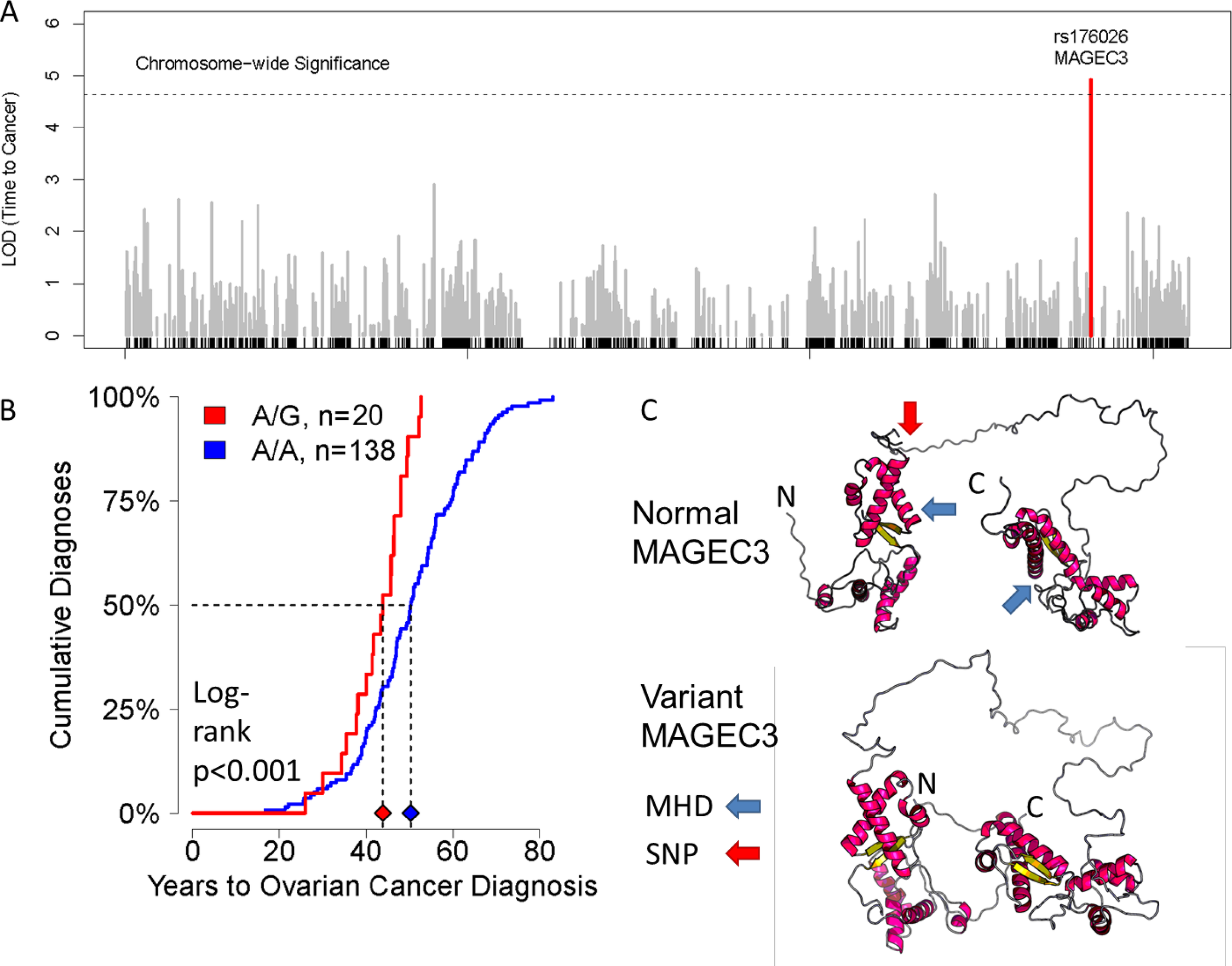

Supplement: S1 Fig — X-chromosome wide exome sequencing (A) yielded a single SNP associated with earlier age of ovarian cancer onset (B). The variant in in MAGEC3 affects the backbone between two MAGE homology domains (C) leading to a predicted conformational change and loss of function. (TIF) [file pgen.1007194.s001.tif]

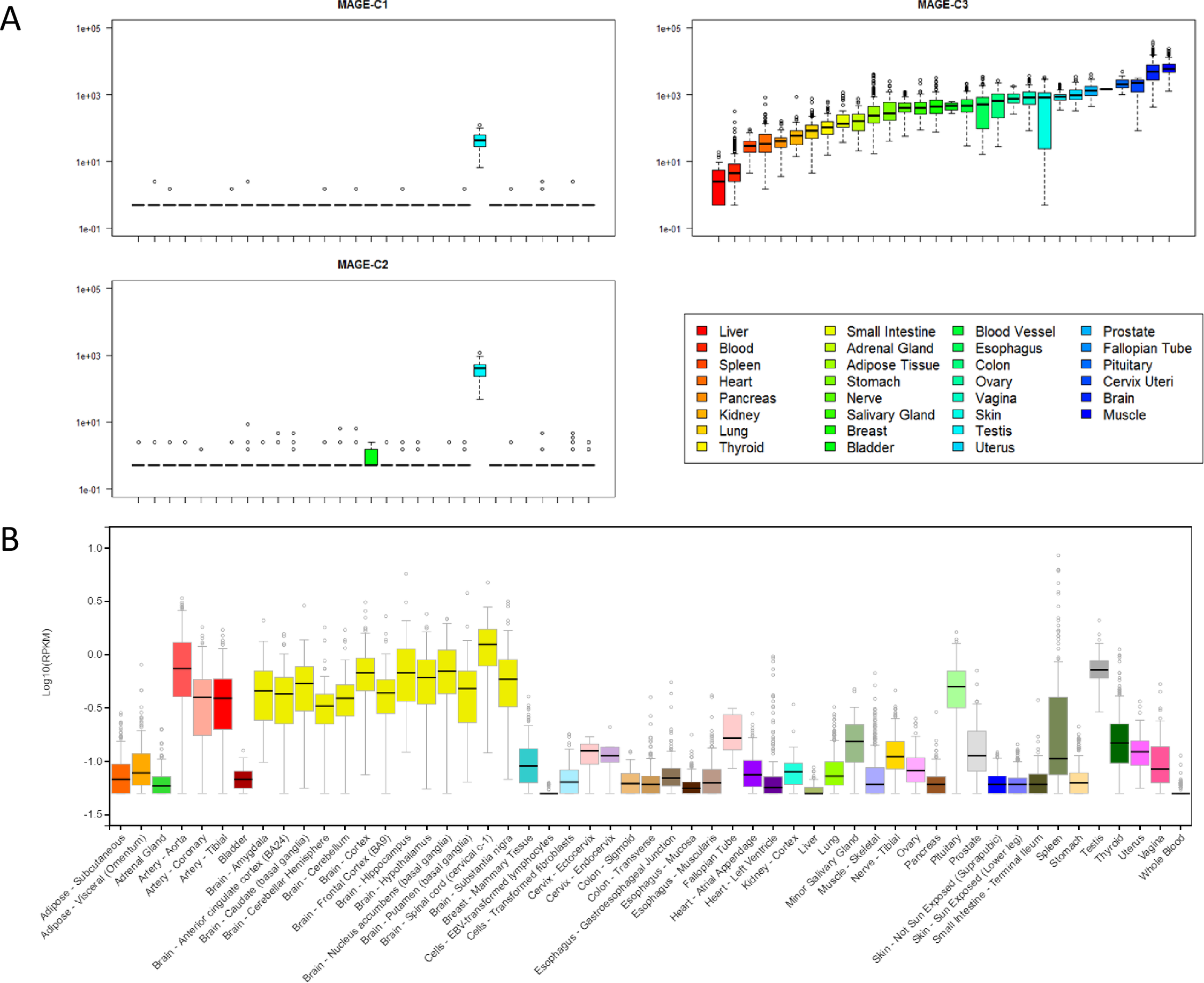

Supplement: S2 Fig — (A) MAGEC1 and MAGEC2 show classic cancer testis antigen patterns while MAGEC3 shows moderate levels of expression in most tissues. (B) Log10 RPKM RNAseq data again shows MAGEC3 has moderate expression in a variety of tissues. (TIF) [file pgen.1007194.s002.tif]

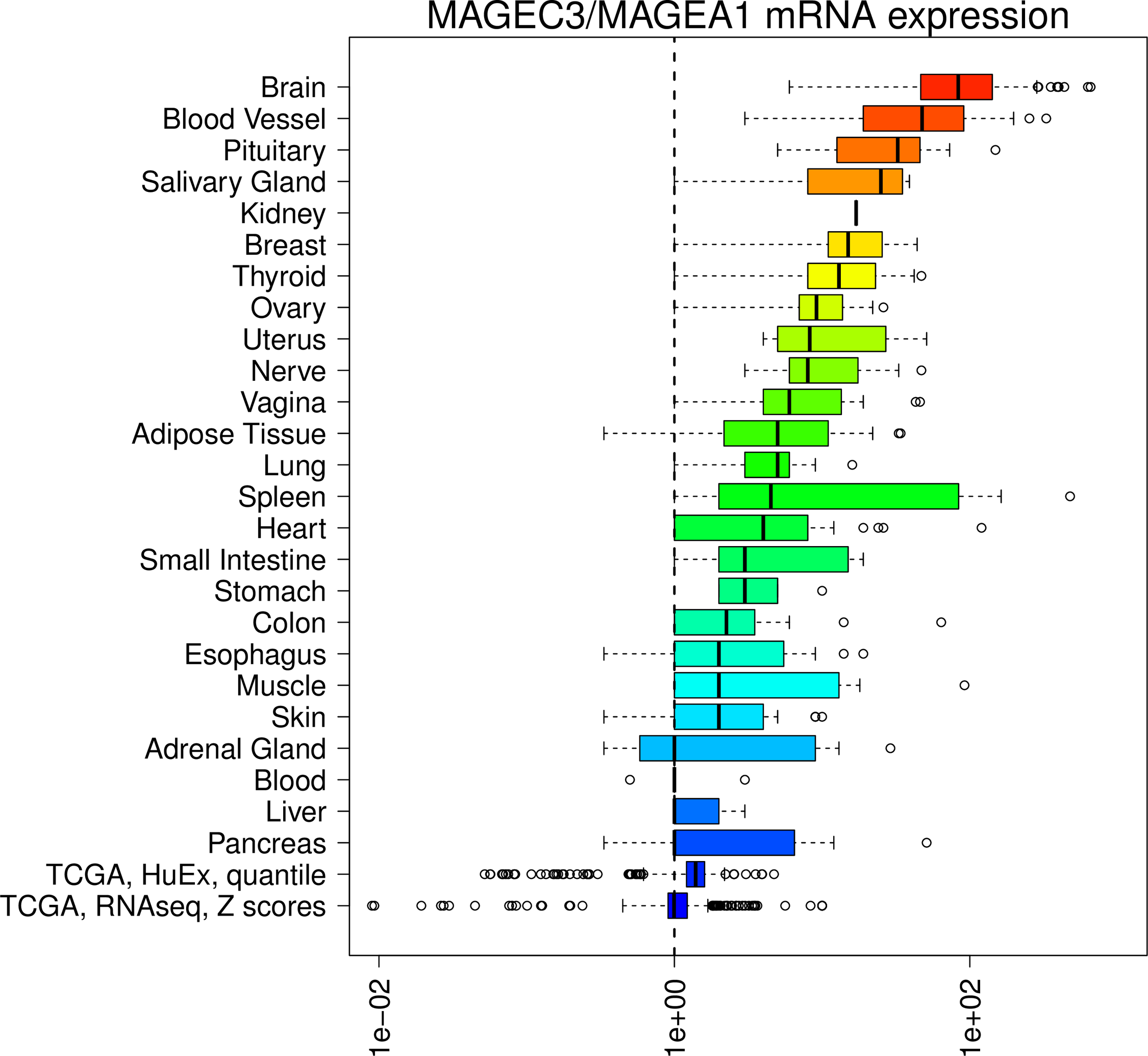

Supplement: S3 Fig — Relative expression to MAGEA1, which is not expressed in normal tissue, is nearly 100x higher in brain tissue. The TCGA categories are ovarian tumors measured by array and RNA sequencing. (TIF) [file pgen.1007194.s003.tif]

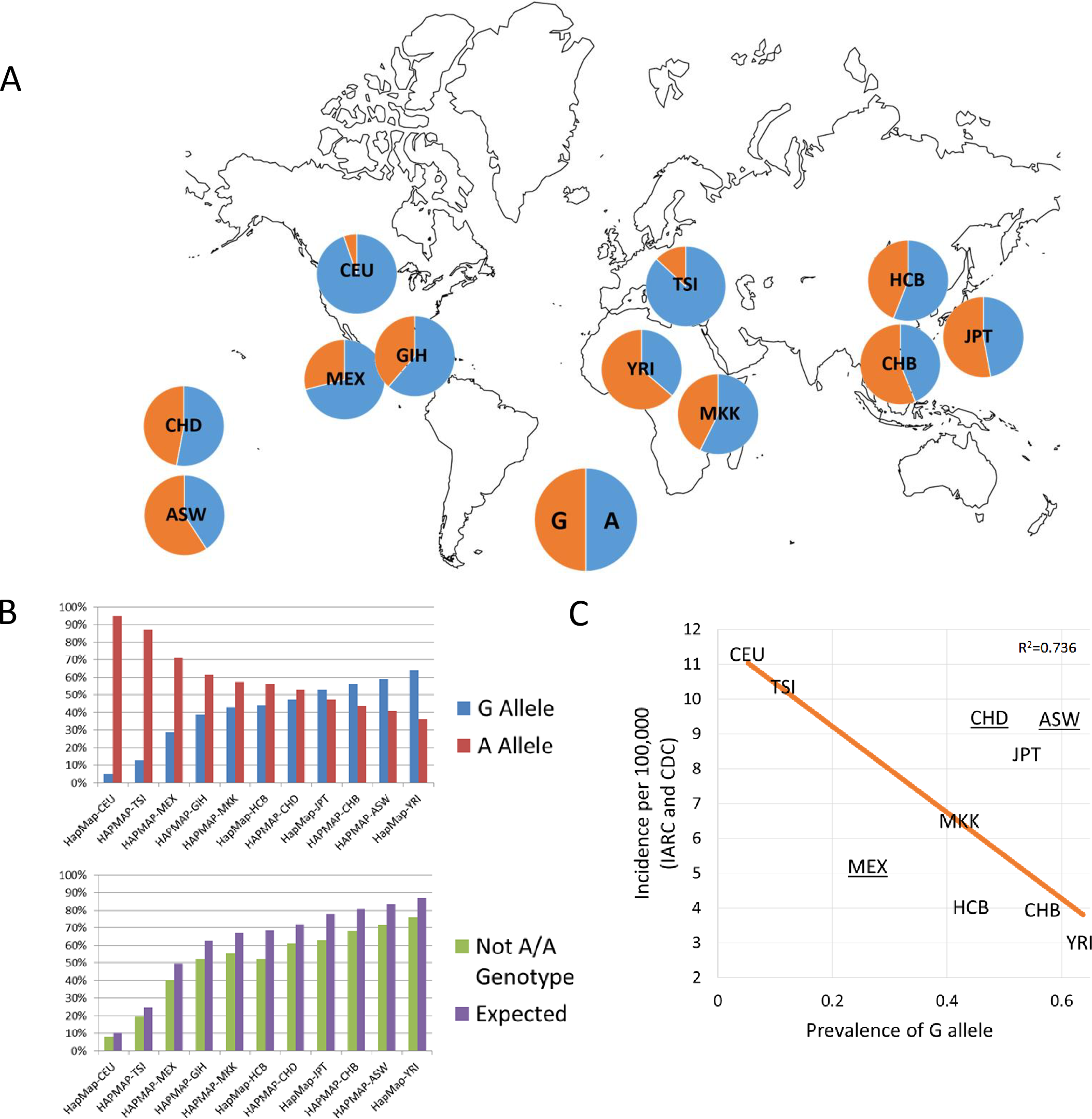

Supplement: S4 Fig — G/A allele relative frequencies by population and geographic location (A); CHD and ASW are the Chinese American and African American populations in Denver and southwest USA. Sorted by allele frequency and expected genotype frequency (B). Alelle frequency is correlated with national incidence of ovarian cancer in HapMap and 1000 genomes (C). (TIF) [file pgen.1007194.s004.tif]
